# Ultrasonic-Assisted extraction of luteolin from peanut shells using supramolecular solvents and its molecular mechanism

**DOI:** 10.1016/j.ultsonch.2025.107678

**Published:** 2025-11-12

**Authors:** Yuhan Fang, Ping Zhang, Shuang Wang, Lulu Li, Zunlai Sheng

**Affiliations:** a College of Veterinary Medicine, Northeast Agricultural University, Harbin 150030, PR China; b Heilongjiang Key Laboratory for Animal Disease Control and Pharmaceutical Development, Harbin 150030, PR China; c Instrumental Analysis Center, Northeast Agricultural University, PR China

**Keywords:** Peanut shells, Luteolin, Supramolecular solvents, Ultrasound-assisted extraction, Formation mechanism

## Abstract

This study innovatively employs ultrasound-assisted extraction (UAE) in conjunction with supramolecular solvents (SUPRAS) for the extraction of luteolin from peanut shells, while also investigating the potential mechanism underlying this extraction process. Initially, the formation mechanism of SUPRAS was characterized using Fourier-transform infrared spectroscopy (FTIR). Based on the results of single-factor experiments, the extraction process was optimized using response surface methodology (RSM), resulting in the determination of optimal conditions: an ultrasound temperature of 67 °C, a SUPRAS/equilibrium solution (EqS) volume ratio of 5:1, and a solid-to-liquid ratio of 1:36 g/mL. Under these conditions, the maximum luteolin yield reached 1.645 mg/g. Furthermore, through independent gradient model (IGMH) calculations, molecular orbital theory, interaction energy analysis, and molecular dynamics simulations, it was revealed that the predominant interaction force between the supramolecular solvent and the target compound is hydrogen bonding. Notably, this study also compares the SUPRAS-UAE extraction method with the traditional extraction in terms of efficiency, economic costs, and environmental impact. The results indicate that the SUPRAS-UAE technique demonstrates significant advantages in terms of its green and high-efficiency characteristics. It provides a new sustainable strategy for the efficient recovery of natural active ingredients in chemical engineering and related fields.

## Highlights

• Combined supramolecular solvents and ultrasound for enhanced luteolin extraction from agricultural waste.
• Hydrogen bonding revealed as the dominant extraction mechanism via IGMH, molecular orbital, and molecular dynamics simulations.
• SUPRAS-UAE outperforms traditional methods in efficiency, cost, and environmental sustainability.
• Provides a chemical engineering strategy for eco-efficient recovery of bioactive compounds from plant matrices.

## Graphical abstract

**Figure:**
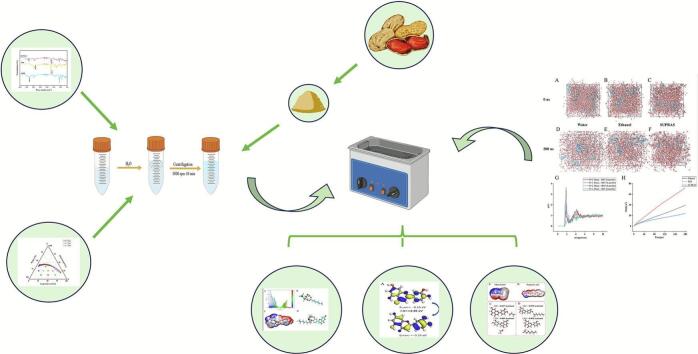

## 1 Introduction

The peanut (Arachis hypogaea) is a significant agricultural crop globally, with China producing over 17 million metric tons of peanuts annually, yielding approximately 5–6 million tons of peanut shells [1]. Traditionally, over 90 % of shells are discarded via incineration or landfilling, causing environmental pollution and resource waste [2]. Notably, peanut shells contain 3.8 %-6.1 % flavonoids—primarily luteolin, eriodictyol, and 5, 7-dihydroxychromone—which demonstrate significant potential for valorisation [3]. Recovering these compounds aligns with circular economy goals and addresses the limitations of synthetic antioxidants linked to hepatotoxicity [4,5]. Among them, luteolin is particularly noteworthy for its significant antioxidant, anti-inflammatory, antibacterial, and anti-cancer activities, making it valuable for various applications in the food, pharmaceutical, and cosmetic industries [6]. The re-use of peanut shells as a source of natural antioxidants not only provides an inexpensive and sustainable material but also offers significant environmental benefits by reducing waste and pollution.

Contemporary methodologies for luteolin extraction include thermal reflux, Soxhlet extraction, enzymatic hydrolysis, subcritical water extraction, and microwave-assisted techniques [[7], [8], [9], [10], [11]]. Current extraction techniques face efficiency and sustainability challenges. While thermal reflux uses ethanolic solutions at elevated temperatures, its practical implementation is complicated by solvent volatility and stringent thermal control requirements [12]. Soxhlet extraction, while enabling thorough solid–liquid interaction, suffers from prolonged operational cycles and excessive solvent consumption [8]. Enzymatic approaches utilizing cellulase-mediated cell wall lysis are limited by narrow pH and temperature tolerance ranges, which compromise enzymatic activity [13]. Subcritical water extraction is still limited to specialized laboratories due to prohibitive infrastructure costs associated with high-pressure systems [14]. Microwave-assisted methods, although advantageous for rapid heating, often induce localized thermal gradients due to uneven electromagnetic field distribution, which can lead to the degradation of thermolabile compounds [15]. These issues highlight the need for novel solvents and energy-efficient processes.

Supramolecular solvents (SUPRAS) offer a green alternative through self-assembled amphiphilic networks, enabling multimodal molecular recognition [16]. The long-chain acid–ethanol-water system developed in this study exemplifies such green innovation through four key attributes: spontaneously segregated polarity microdomains enabling multimodal molecular recognition via hydrophobic π-π/dispersion interactions and hydrophilic hydrogen/ion–dipole bonding [17]; high amphiphile concentrations create dense binding site networks with rapid interfacial mass transfer [18]; environment-independent self-assembly facilitates simple preparation [19]; and full-component biodegradability (Long-chain acid, ethanol, water) aligning with the 12 Principles of Green Chemistry. While SUPRAS have demonstrated efficacy in environmental contaminant analysis, their application in the recovery of plant-derived bioactive compounds remains underexplored, particularly regarding the microstructural analysis of the composite system and the underlying extraction mechanism, which warrants further investigation.

This study pioneers a SUPRAS system based on heptanoic acid–ethanol-water, overcoming toxicity concerns of conventional formulations. When integrated with ultrasonic-assisted extraction (UAE), SUPRAS leverages microdomain polarity segregation for selective luteolin capture, while UAE enhances mass transfer via cavitation effects, reducing extraction time from hours to minutes and boosting yield compared to ethanol reflux [20]. The novelty of this work encompasses three dimensions: (a) A biodegradable SUPRAS formulation replacing tetrahydrofuran, optimized via RSM for luteolin selectivity. (b) Mechanistic insights: Synchronized FT-IR and fluorescence microscopy reveal in situ solvent self-assembly kinetics, while IGMH and FMO (frontier molecular orbital) analyses quantify solute–solvent interactions at atomic resolution. (c) Process benchmarking: The SUPRAS-UAE protocol achieves an extraction efficiency of over 95 % with 60 % lower carbon emissions compared to conventional methods. This study establishes a sustainable paradigm for valorizing agro-industrial waste, merging supramolecular chemistry with process engineering to advance bioactive compound recovery.

## 2 Materials and methods

### 2.1 Materials and instruments

Fresh peanuts (cultivar: Da Bai Sha) were procured from a certified supplier, and the shells were carefully separated using stainless-steel tools. The shells were dehydrated in a convection oven at 60 ± 2°C for 24 h until reaching a stable weight (Δm < 0.1 % per hour). The dried shells were pulverized using a stainless-steel grinder, passed through a 60-mesh screen, and subsequently preserved in polyethylene pouches for subsequent experimental applications.

Luteolin (certified purity ≥ 98 %, batch code: L107329) was obtained from Shanghai Maclin Biochemical Technology Co., Ltd. (Shanghai, China). Heptanoic acid (Hea, ≥ 98 %), octanoic acid (Oca, ≥ 98 %), nonanoic acid (Noa, ≥ 98 %), and fluorescein sodium salt (≥99 %) were sourced from Aladdin Industrial Corporation (Shanghai, China). Acetonitrile (chromatographic grade) and formic acid used in this study were purchased from Kemio Chemical Reagents Co., Ltd. (Tianjin). Deionized water was produced using a Milli-Q Academic water purification system, while all other analytical-grade reagents were supplied by Shanghai Aladdin Biochemical Technology Co., Ltd.

Sonochemical processing in this study was conducted using an ultrasonic processor (Model KQ-250DB, Kunshan, Jiangsu, China) equipped with a 250 W power supply. This apparatus incorporates a rectangular reaction chamber with internal dimensions of 23.5 cm (length) × 13.3 cm (width) × 10.3 cm (height). Annealed piezoelectric transducer arrays, operating at an operational frequency of 50 kHz, were strategically mounted at the base of the tank to ensure homogeneous acoustic energy distribution. High-performance liquid chromatography (HPLC) analysis of flavonoids from peanut shell extracts was performed using a Shimadzu LC-20AR system (Shimadzu International Trading Co., Ltd.), equipped with a 7725i manual injector, binary gradient pump, and SPD-20UV detector, ensuring high-resolution separation of multicomponent systems in complex matrices. For the identification and confirmation of the target compound, an analysis was conducted using a Waters liquid chromatography-mass spectrometry (LC-MS) system comprised of a 2695 separations module, a 2996 photodiode array (PDA) detector, and a ZQ2000 mass spectrometer with an electrospray ionization (ESI) source.

### 2.2 Synthesis and characterization of SUPRAS

#### 2.2.1 Synthesis of SUPRAS

Initially, amphiphilic reagents (Hea, Oca, Noa) were introduced into a 50 mL conical tube at defined molar ratios with ethanol. Deionized water was delivered via a peristaltic pump as a coacervate-inducing agent until the ternary system reached a volume of 40.0 ± 0.1 mL. The system was vortex-mixed for 1 min to initiate supramolecular self-assembly. Phase separation was achieved by centrifugation at 5000 × g for 10 min (25 °C), resulting in the SUPRAS as the upper phase and the equilibrium solution (EqS) as the lower phase. Both the upper and lower phases (SUPRAS and EqS) were separately collected and stored under sealed conditions at 4 °C.

#### 2.2.2 Phase diagram

The ternary phase diagram of the long-chain acid–ethanol–water system was constructed using cloud-point titration under isothermal conditions [21]. Initially, 100 μL of ethanol was mixed with 1000 μL of long-chain acid to form a homogeneous solution. Deionized water was gradually added to the solution until turbidity (i.e., cloud point) appeared, and the exact volume of added water was recorded. Subsequently, 100 μL of ethanol was added to the mixture, leading to the clearance of turbidity. Deionized water was then incrementally added until the turbidity was restored, and the volumes of long-chain acid, ethanol, and water were accurately recorded. This procedure was repeated to obtain sufficient data for constructing the phase diagram. The ternary phase diagram was generated by plotting the volume percentages of each component at the onset of turbidity.

#### 2.2.3 SUPRAS formation mechanism

The synthesis mechanism and molecular interactions were investigated using Fourier Transform Infrared (FT-IR) spectroscopy. A Nicolet iS50 spectrometer (Thermo Fisher Scientific, USA) was employed to analyze the individual components of SUPRAS as well as the final SUPRAS sample, with spectra collected in the range of 4000–400 cm^−1^ at a resolution of 0.09 cm^−1^.

### 2.3 Determination of luteolin content in Peanut shells

#### 2.3.1 Preparation of the standard curve for luteolin

Luteolin quantification was performed using a calibration curve constructed according to the analytical protocol reported by Giacometti et al.[22]. A stock solution of luteolin was prepared by dissolving 10.0 mg of accurately weighed luteolin standard in methanol and diluting to a final volume of 10 mL. Working solutions at concentrations of 0.01, 0.03, 0.05, 0.07, and 0.09 mg/mL were generated via serial dilution: aliquots of 0.1, 0.3, 0.5, 0.7, and 0.9 mL of the stock solution were transferred into individual 10 mL volumetric flasks and brought to volume with methanol. All solutions were homogenized before analytical measurements. The calibration curve for luteolin (R^2^ = 0.9995) spanned the concentration range of 0.01–0.09 mg/mL, with the regression equation defined as (1):

(1) $$y=7.0\times 10^{7}x-74091$$

where x  = analyte concentration (mg/mL) and y = corresponding peak area.

#### 2.3.2 Extraction program

**Fig. 1:**
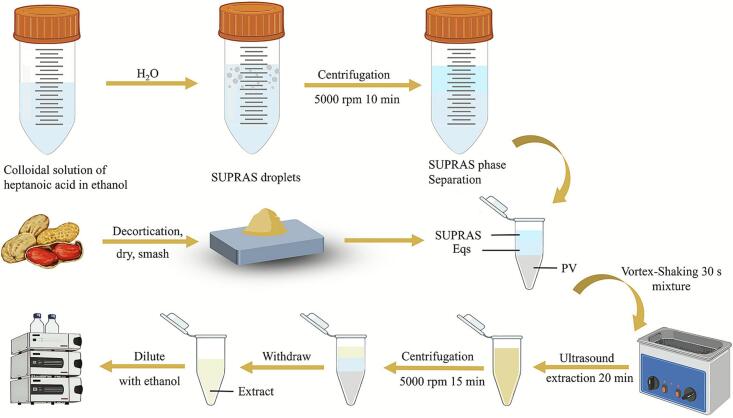
Schematic picture of the SUPRAS/EqS extraction process of luteolin.

Precisely 0.2 mg of the powdered sample was homogenized with specific volume ratios of SUPRAS and EqS to achieve a total system volume of 6.0 mL (Fig. 1). The homogenate was vortexed for 30 s. Then it was processed via ultrasonic treatment (60 °C, 100 W) for a 20-minute duration. After extraction, the mixture was stabilized at room temperature before being centrifuged at 5000 × g for 15 min. The supernatant was quantitatively transferred and diluted with anhydrous ethanol for content analysis.

#### 2.3.3 Determination of luteolin content

The quantitative determination of luteolin was performed using HPLC (Shimadzu 2010 AR system) equipped with a C18 Diamonsil reversed-phase column (250 mm × 4.6 mm, 5 μm particle size). The isocratic elution method involved a mobile phase system consisting of acetonitrile (phase B) and 0.1 % formic acid in water (phase A) at a volumetric ratio of 30:70, delivered at 1.0 mL/min with the column temperature maintained at 30 °C. Detection was carried out using an SPD-20 UV–Vis detector set at the characteristic wavelength of 350 nm, with a 20 μL injection volume. The luteolin content was determined by interpolating the peak area values from the calibration curve (Section 2.2) and was calculated according to Eq. (2):

(2) Yield=c×v/M(mg/g)

where c represents the luteolin concentration (mg/mL), v denotes the solution volume (mL), and M corresponds to the sample mass (g).

### 2.4 Optimization of the extraction program

#### 2.4.1 Single-factor experiment

To establish the optimal extraction process, this study systematically optimized the key operational parameters, including ultrasonic treatment time (10 min, 15 min, 20 min, 25 min, 30 min), ultrasonic temperature (30 °C, 40 °C, 50 °C, 60 °C, 70 °C, 80℃), ultrasonic power (150 W, 175 W, 200 W, 225 W, 250 W), the SUPRAS: EqS ratios (1:0, 1:2, 1:1, 2:1, 5:0), and the liquid-to-solid ratio (LSR) (18, 24, 30, 36, 42 mL/g). The optimization of these parameter windows was aimed at constructing a process framework that enhances extraction efficiency.

#### 2.4.2 RSM

Building on the outcomes from the single-factor experiments, this study utilized the Box-Behnken Design (BBD) within the framework of RSM to develop the experimental design, analyze the results, and create a regression model. Luteolin content was selected as the response variable, with ultrasound temperature, SPURAS: Eqs ratio, and LSR as independent variables (Table 1). A total of 17 experimental runs were conducted using Design Expert software, incorporating the interactions of three factors (ultrasound temperature, SPURAS: Eqs, and LSR) at three different levels. Each experimental combination was replicated three times to ensure the reliability and consistency of the results.

**Table 1:** Coding of experimental parameters and related levels.

Experimental	Unit	Symbols			Coded values
parameters		(X_i_)	Low (−1)	Medium (0)	High (+1)
Ultrasonic temperature	℃	X1	60	70	80
SUPRAS: Eqs	(v/v)	X2	2:1	5:1	6:0
Liquid-solid ratio	mL/g	X3	30	36	42

### 2.5 Extraction mechanism study

#### 2.5.1 DFT calculation

Molecular geometry optimization was conducted at the B3LYP/6-31G(d) level using the Gaussian 16 W software suite, with structural visualization provided by GaussView 6.0. The subsequent characterization of the electronic structure involved detailed analyses of molecular electrostatic potentials using Multiwfn 3.8, complemented by isosurface topology mapping and charge distribution visualization on molecular surfaces using VMD, thereby elucidating the compound's electronic properties. Furthermore, to investigate the supramolecular extraction mechanism in greater detail, computational simulations were conducted using Materials Studio 2019 (MS). Fukui functions and frontier molecular orbitals were systematically analyzed to identify nucleophilic and electrophilic reactive sites. Additionally, within the Forcite module, the steepest descent and conjugate gradient methods were employed to geometrically optimize the structures of both the target molecule and the extracted solvent, identifying the two most stable binding modes and calculating the binding energy of each mode. The interaction energy between the two molecules is obtainable through the subsequent equation [23](3):

(3) $$E_{\mathrm{int}}=E_{\mathrm{cluster}}-\left(E_{A}+E_{B}\right)$$

Where *E_int_* refers to the interaction energy, *E_cluster_* is the total energy of the molecular complex formed by luteolin and SUPRAS, and *E_A_* and *E_B_* represent the energies of the isolated molecules A and B, respectively.

#### 2.5.2 Molecular dynamics simulation

Molecular dynamics simulations were performed using Materials Studio 2023 software to investigate the extraction mechanism of luteolin with SUPRAS. The molecular models of Hea, ethanol, water, and luteolin were initially constructed and geometrically optimized using the Forcite module with COMPASS III force field assignment. Atomic charges were calculated through the charge equilibration method. Three distinct amorphous cell (AC) systems were subsequently established using the Amorphous Cell module: (a) SUPRAS-luteolin system (47.23 Å^3^), (b) ethanol-luteolin system (46.83 Å^3^), and (c) water-luteolin system (47.24 Å^3^). Each system underwent energy minimization before dynamic simulations. The equilibration protocol consisted of two sequential phases: initial stabilization under the NVT ensemble (50 ps, Berendsen thermostat) followed by production runs under the NPT ensemble (500 ps, Andersen thermostat). Trajectory data were recorded at 1 ps intervals for subsequent analysis of molecular interactions and system dynamics. All simulations maintained constant temperature (298 K) and pressure (1 atm) conditions with periodic boundary conditions.

### 2.6 Compared with traditional methods

#### 2.6.1 Comparison of environment and efficiency

This investigation systematically evaluated two luteolin extraction methodologies from peanut hulls. Ultrasonic-assisted water extraction (Water-UAE) processed 0.2 g of dried sample with 7.2 mL of deionized water at 250 W for 15 min, with subsequent filtration and concentration before HPLC quantification as described in Section 2.2.4. Alternatively, thermal reflux extraction using 70 % (v/v) ethanol (ER) processed 1 g of powdered material with 36 mL of solvent for 2 h, followed by identical post-treatment. Notably, based on experimental outcomes, the environmental impact and economic costs of both methods were comparatively analyzed through extraction yield, process efficiency, energy economy, and carbon footprint mitigation, enabling a holistic evaluation of the practical applicability of the supramolecular extraction technology.

#### 2.6.2 Scanning electron microscopy analysis

Surface morphological features of peanut hulls subjected to three distinct pretreatment protocols were characterized using a tungsten-filament scanning electron microscope (SEM) operated at 15 kV. The investigated samples comprised: (A) air-dried hulls processed via ultrasonic extraction in an aqueous medium, (B) specimens treated with 70 % (v/v) ethanolic thermal reflux extraction, and (C) matrices undergoing SUPRAS-mediated ultrasonication. Microstructural evaluation was conducted at magnifications of 500 × and 1000 × for all air-dried specimens.

### 2.7 The LC-MS analysis of peanut shell extract

Chromatographic separation was achieved on a Waters Sunfire C18 column (4.6 × 100 mm, 3.5 μm) maintained at 30 °C. The mobile phase consisted of 0.1 % formic acid in water (phase A) and 0.1 % formic acid in acetonitrile (phase B), delivered at a constant flow rate of 0.8 mL/min with an injection volume of 10 μL. The gradient elution program was as follows: 5 % B (0.00 min) → 95 % B (7.00 min) → 95 % B (12.00 min) → 5 % B (12.20 min) → 5 % B (15.00 min). Detection was performed using the photodiode array (PDA) detector at 350 nm.

For mass spectrometric detection, an electrospray ionization (ESI) source was operated in full scan mode over the range of *m*/*z* 50–1500. The optimized parameters were as follows: source temperature, 140 °C; desolvation temperature, 350 °C; capillary voltage, 3.0 kV; cone voltage, 10 V; extractor voltage, 5 V; RF lens voltage, 0.3 V. The desolvation and cone gas flow rates were set at 800 L/h and 50 L/h, respectively.

### 2.8 Statistical analysis

To ensure data reliability and reproducibility, all extraction experiments were conducted in triplicate. Experimental data were statistically analyzed using Design Expert 13 software, with results expressed as mean ± standard deviation (SD). One-way ANOVA was performed to evaluate inter-group differences, with significance defined as *p* < 0.05 (95 % confidence interval). RSM was employed to develop a multivariate regression model, enabling the interrogation of interactive effects among critical parameters on extraction efficiency. Three-dimensional response surface plots were generated to identify optimal extraction conditions.

## 3 Results and Discussion

### 3.1 SUPRAS synthesis and phase diagram

**Fig. 2:**
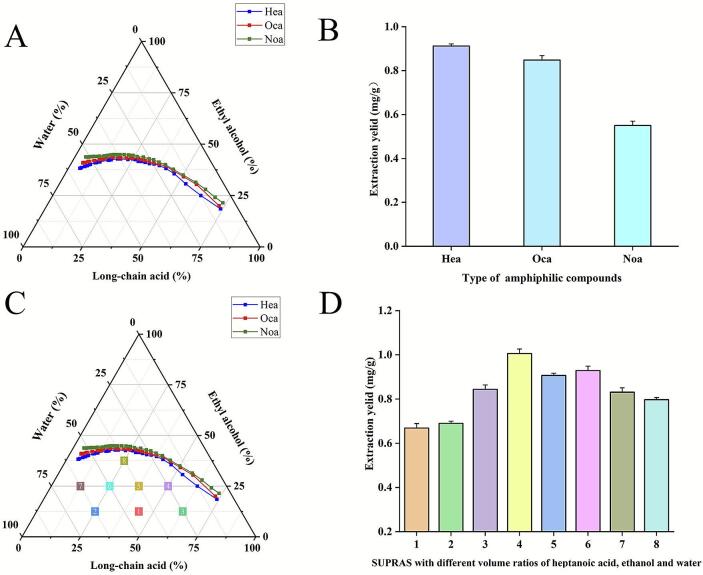
(A) phase diagrams of SUPRAS prepared with different long-chain acids. (B) The extraction yield of different SUPRAS. (C) The SUPRAS phase diagram formed by Hea, ethanol and water and the effect of SUPRAS composition on the extraction yield. In Fig. 2c, the ratios of Hea: ethanol: water were as follows: (1) 7:2:7, (2) 2:1:5, (3) 5:1:2, (4) 2:1:1, (5) 3:2:3, (6) 1:1:2, (7) 1:2:5, and (8) 2:3:3. (D) the extraction yield of different SUPRAS compositions.

This study systematically investigated the formation patterns of SUPRAS by constructing ternary phase diagrams for amphiphilic compounds with varying hydrophobic chain lengths (Hea, Oca, and Noa) in ethanol/water systems. The phase diagrams (Fig. 2A) revealed two distinct regions: a homogeneous monophasic domain above the binodal curve and a well-defined SUPRAS formation zone below. Experimental results demonstrated progressive expansion of the SUPRAS formation domain with increasing hydrophobic chain length. This phenomenon can be attributed to the fact that as the carbon chain length of amphiphilic compounds increases, their polarity strengthens. Conversely, reduced polarity favors the attainment of the critical micelle concentration in isotropic solutions, consequently boosting the energetic driving force for molecular self-aggregation. This, in turn, promotes the directed formation of ordered SUPRAS [24].

Based on the results from the ternary phase diagram, under the condition where the volume ratio of long-chain acid/ethanol/water is fixed at 3:2:3 within the stable SUPRAS formation zone, SUPRAS systems were constructed using Hea, Oca, and Noa. Fig. 2B demonstrates that the SUPRAS formed from Hea exhibits the optimal extraction performance (0.92 ± 0.01 mg/g), while the extraction capacities of the Oca (0.84 ± 0.02 mg/g) and Noa (0.55 ± 0.02 mg/g) systems decrease progressively with the elongation of the carbon chain. Previous studies have indicated that an increase in carbon chain length can enhance extraction efficiency by strengthening hydrophobic interactions [25]. However, this study reveals that excessive elongation of the alkyl chain leads to a decrease in extraction performance. This finding aligns with the research conducted by Dingli Ye et al [26], who concluded that both viscosity and hydrophobicity play critical roles in determining extraction efficiency, with increasing viscosity negatively impacting the extraction and transfer of the target product. Consequently, Hea was selected for further experiments.

To investigate the composition-dependent extraction efficiency of luteolin in SUPRAS systems, eight characteristic phase points, with varying Hea/ethanol/water volume ratios, were systematically examined within the stable SUPRAS region using ternary phase diagram analysis (Fig. 2C). Experimental data results are shown in Fig. 2D. This phenomenon can be attributed to the following factors: (i) The proportion of long-chain acid determines SUPRAS polarity. Increased polarity enhances hydrogen bonding and polar interactions, facilitating the dissolution of hydrophilic compounds, while reduced polarity strengthens dispersive interactions for hydrophobic species [27]; (ii) The ethanol content exhibits dual regulatory mechanisms: moderate reduction enhances hydrophobic interactions, while excessive depletion increases the viscosity of SUPRAS, thereby impeding mass transfer kinetics [28]. Conversely, higher ethanol levels (e.g., Phase Point 8) induce a phase partitioning disequilibrium, leading to a decrease in extraction efficiency [29]. By optimizing the amphiphile-ethanol synergism, the optimal volume ratio was determined to be Hea: ethanol: water = 2:1:1 (Phase Point 4).

### 3.2 SUPRAS formation mechanism

**Fig. 3:**
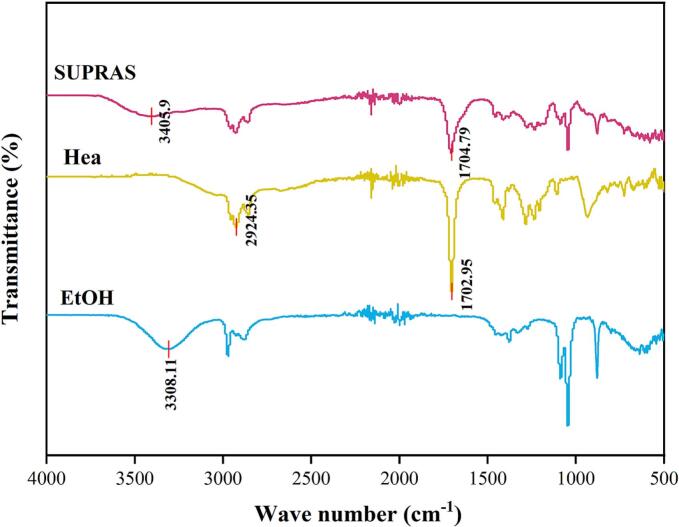
FTIR spectra of heptanoic acid, ethanol, SUPRAS.

FTIR was employed to characterize the synthesized SUPRAS and its individual components, Hea and ethanol (Fig. 3). As a monocarboxylic acid, Hea forms a dimer structure through intermolecular hydrogen bonding, with its –OH stretching vibration showing a redshift due to hydrogen-bond association, exhibiting a characteristic peak at 2927 cm^−1^. The –OH vibration peak of ethanol appears at 3304 cm^−1^, with no new peaks detected, indicating that no chemical reactions occurred during the synthesis of SUPRAS. In the SUPRAS system, the –OH vibration peak shifted further to 3310 cm^−1^, and the intensity of the peak significantly increased and broadened compared to the Hea monomer. This phenomenon suggests the potential formation of a synergistic hydrogen-bond network between Hea and ethanol. Furthermore, the C=O characteristic peak of Hea exhibited considerable broadening after the formation of SUPRAS, indicating the further development of intermolecular hydrogen bonds and the formation of reverse micelles [30].

It should be noted that the O-H bond of liquid water exhibits vibrational absorption around 3200–3400 cm^−1^. In the SUPRAS system, water, as a key component, participates in the construction of the hydrogen-bond network. The O-H bond of water molecules can form multiple hydrogen-bond interactions with the carboxyl group (–COOH) of Hea and the hydroxyl group (–OH) of ethanol. which is of great significance for the structural stability of the SUPRAS system and the subsequent interaction with luteolin during the extraction process.

### 3.3 Optimization results of the single-factor extraction conditions

#### 3.3.1 Effect of ultrasonic extraction time on luteolin content

**Fig. 4:**
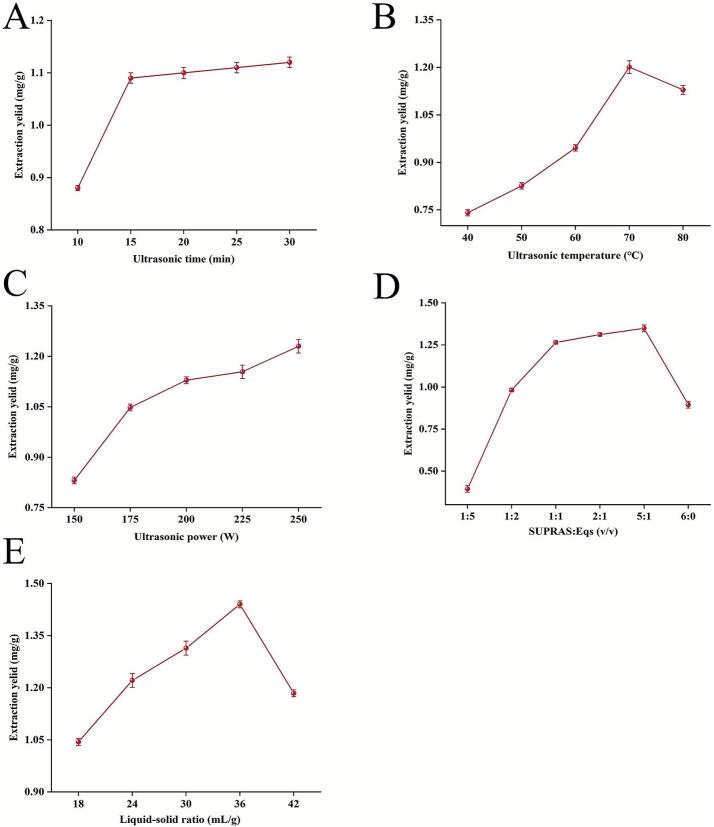
The influence of different factors on the extraction rate of luteolin from peanut shells. (A) ultrasound time, (B) ultrasonic temperature, (C) ultrasonic power, (D) SUPRAS: Eqs, (E) liquid–solid ratio.

Ultrasound-induced cavitation effectively disrupts plant cell walls, enhancing the dissolution of the target compound [31]. The critical parameters of ultrasound-assisted extraction were systematically investigated to optimize luteolin extraction. As shown in Fig. 4A, extending the sonication duration from 10 to 30 min increased luteolin yield from 0.89 ± 0.02 mg/g to 1.12 ± 0.03 mg/g. Notably, the extraction yield plateaued at 1.09 ± 0.04 mg/g–1.12 ± 0.03 mg/g once the sonication duration reached 15 min. This stabilization is attributed to cavitational microjets generated by bubble collapse, which overcome mass transfer resistances, achieving complete compound liberation. Once solvent permeation reached equilibrium, further sonication had little effect on solute migration [32]. Therefore, 15 min was established as the optimal sonication time, ensuring maximum extraction efficiency while minimizing energy use in line with green chemistry principles.

#### 3.3.2 Effect of ultrasonic temperature on luteolin content

Ultrasonic temperature, as a critical thermodynamic parameter, significantly influences the extraction kinetics by modulating the intensity of cavitation effects and the diffusion rate of molecules [33]. As illustrated in Fig. 4B, when the temperature was increased from 40 °C to 70 °C, the luteolin yield rose from 0.74 ± 0.01 mg/g to 1.20 ± 0.03 mg/g. This enhancement is attributed to the elevated temperature, which intensifies the microjet strength generated by cavitation bubble collapse, thereby increasing the solute diffusion coefficient and accelerating the desorption of target compounds from the matrix [34]. However, when the temperature exceeded 70 °C, the yield declined to 1.12 ± 0.02 mg/g (at 80 °C), likely due to excessive cavitation leading to the degradation of heat-sensitive compounds or intensified solvent evaporation, resulting in a loss of effective volume [35]. Therefore, 70 °C was selected as the optimal balance point, where the efficiency of ultrasonic energy conversion and the stability of the target compound are maximized in synergy.

#### 3.3.3 Effect of ultrasonic power on luteolin content

Sonication power significantly governs extraction dynamics through its modulation of cavitation energy density. As evidenced in Fig. 4C, escalating power input from 150 W to 250 W induced a non-linear ascending profile in the yield of luteolin, increasing from 0.83 ± 0.01 mg/g to 1.24 ± 0.04 mg/g. This progression is mechanistically attributable to power-dependent amplification of acoustic pressure amplitude, intensified mechanical energy release during transient bubble collapse effectively disrupts plant tissue ultrastructure [36]. Given the sustained yield augmentation observed at the instrumental maximum (250 W), this power level was selected for subsequent experimental protocols to optimize extraction efficiency.

#### 3.3.4 Effect of SUPRAS: Eqs ration on luteolin content

To elucidate the mechanistic impact of the SUPRAS-to-EqS phase ratios on the selective extraction of target compounds, the synergistic effects of solvent composition were systematically explored. The Fig. 4D revealed a marked ascending gradient in target compound yield as the SUPRAS: EqS volumetric ratio increased from 1:5 to 5:1, with peak efficiency attained at the 5:1 ratio. This phenomenon can be attributed to the optimal proportion of EqS, which enhances matrix permeability by improving solvent wetting efficiency and selectively eluting polar interferents [37]. “Notably, when the dominance of SUPRAS exceeded the 5:1 threshold, it inversely affected extraction efficiency, resulting in a reduced yield (0.89 ± 0.02 mg/g) in the pure SUPRAS system (1:0). Consequently, the 5:1 SUPRAS: EqS ratio was selected as the optimal extraction condition.

#### 3.3.5 Effect of liquid–solid ratio on luteolin content

The LSR significantly influenced the extraction process by modulating the efficiency of solvent-substrate contact [38]. As shown in Fig. 4E, the luteolin yield progressively increased with an elevation in the LSR from 18 to 36 mL/g, reaching a maximum value of 1.48 ± 0.01 mg/g. This trend can be attributed to the enhanced solvent permeability, which improves the dissolution of the target compound from the peanut shell matrix at moderate LSR levels [39]. However, beyond the 36 mL/g threshold, excessive solvent volume likely caused analyte over-dilution and reduced the mass transfer driving force, leading to a decline in yield. These findings demonstrate that an LSR of 36 mL/g achieves an optimal balance between solvent utilization efficiency and extraction kinetics.

### 3.4 Optimization of the extraction of luteolin using SUPRAS via RSM

#### 3.4.1 Model Fitting and statistical analysis

A three-factor, three-level Box-Behnken design (BBD) was employed to systematically optimize the critical operational parameters governing the extraction process. Sonication temperature (A), the SUPRAS-to-EqS ratio (B), and the liquid-to-solid ratio (C) were selected as independent variables, generating a response surface matrix consisting of 17 experimental runs (Table 2), created using Design-Expert 13.0 software (Version 13.0, Stat-Ease Inc., USA). Five replicated center points were included to assess experimental reproducibility, followed by statistical evaluation of the model through variance analysis. The obtained second-order polynomial equation is given below (4):

**Table 2:** List of experimental values and predicted values from RSM.

Run	Independent variables			The yield of luteolin (mg/g)	
	Ultrasonic temperature (℃)	SUPRAS: Eqs (v/v)	Liquid-solid ratio (mL/g)	Actual values	RSM predicted
1	0	0	0	1.6	1.61
2	0	1	−1	1.22	1.19
3	0	0	0	1.62	1.61
4	0	0	0	1.57	1.61
5	0	0	0	1.63	1.61
6	0	−1	1	1.09	1.12
7	0	1	1	1.5	1.53
8	1	0	1	1.35	1.33
9	0	0	0	1.62	1.61
10	−1	1	0	1.46	1.46
11	0	−1	−1	1.25	1.22
12	−1	0	1	1.46	1.42
13	−1	−1	0	1.04	1.05
14	−1	0	−1	1.03	1.05
15	1	1	0	1.41	1.4
16	1	−1	0	1.42	1.42
17	1	0	−1	1.42	1.46

Y = 1.608 + 0.0763*X_1_* + 0.0988*X_2_* + 0.06*X_3_* − 0.1075*X_1_X_2_* + 0.125*X_1_X_3_ +* 0.11*X_2_X_3_* − 0.1128*X_1_^2^* − 0.1628*X_2_^2^* − 0.1802*X_3_^2^* (4).

The statistical evaluation of the regression model (ANOVA) is detailed in Table 3. ANOVA revealed significant model performance (*p* < 0.0001), with the coefficient of determination R^2^ = 0.985 and an adjusted R^2^ = 0.966, indicating that the model accounts for 96.6 % of the response variability. All linear terms (*X_1_*, *X_2_*, *X_3_*), interaction terms (*X_1_X_2_*, *X_1_X_3_*, *X_2_X_3_*), and quadratic terms (*X_1_^2^*, *X_2_^2^*, *X_3_^2^*) showed statistical significance (*p* < 0.05), confirming complex nonlinear interdependencies between the process variables and extraction efficiency.

**Table 3:** ANOVA for Quadratic model.

**Source**	Sum of Squares	df	Mean Square	F-value	p-value	
**Model**	0.6461	9	0.0718	50.73	< 0.0001	**Significant**
**X_1_-Ultrasonic temperature**	0.0465	1	0.0465	32.87	0.0007	
**X_2_-SUPRAS: Eqs**	0.078	1	0.078	55.13	0.0001	
**X_3_-Liquid-solid ratio**	0.0288	1	0.0288	20.35	0.0028	
**X_1_X_2_**	0.0462	1	0.0462	32.67	0.0007	
**X_1_X_3_**	0.0625	1	0.0625	44.17	0.0003	
**X_2_X_3_**	0.0484	1	0.0484	34.2	0.0006	
**X_1_^2^**	0.0535	1	0.0535	37.83	0.0005	
**X_2_^2^**	0.1115	1	0.1115	78.82	< 0.0001	
**X_3_^2^**	0.1368	1	0.1368	96.68	< 0.0001	
**Residual**	0.0099	7	0.0014			
**Lack of Fit**	0.0076	3	0.0025	4.46	0.0914	**Not significant**
**Pure Error**	0.0023	4	0.0006			
**Cor Total**	0.656	16				

#### 3.4.2 RSM analysis

**Fig. 5:**
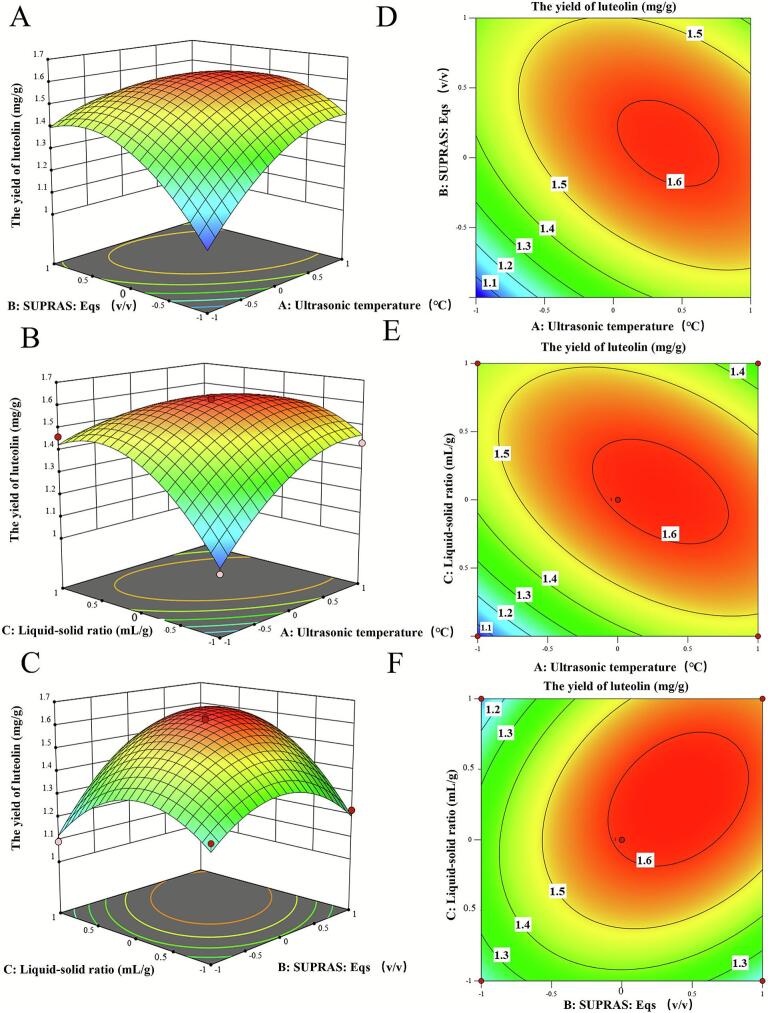
3D response surface graph (left) and contour plot (right) reflect the interactive effects of extraction parameters on the yield of luteolin from peanut shells: ultrasonic temperature and SUPRAS: Eqs (A, D); ultrasonic temperature and liquid–solid ratio (B, E); SUPRAS: Eqs and liquid–solid ratio (C, F).

The three-dimensional response surfaces and corresponding two-dimensional contour plots provide visual evidence of the synergistic interplay between operational parameters. In Fig. 5A-F indicated substantial individual effects of sonication temperature (*X_1_*), the SUPRAS-to-EqS ratio (*X_2_*), and liquid-to-solid ratio (*X_3_*) on extraction efficiency. Additionally, the elliptical isopleths demonstrate significant interactive effects between *X_1_ X_2_*, *X_1_ X_3_*, and *X_2_ X_3_* [40]. These findings align with the data presented in Table 3 and collectively demonstrate that the proposed methodology is reliable, accurate, and capable of effectively predicting extraction performance.

The optimization results of the model indicated that the optimal extraction conditions for the target compound were as follows: ultrasonic temperature of 67.54 °C, SUPRAS/ EqS volume ratio of 5.16: 0.84, and liquid-to-solid ratio of 35.94 mL/g, with a theoretically predicted yield of 1.64 mg/g. Based on practical operation, the parameters were adjusted to 67 °C, 5:1, and 36 mL/g. The verification experiments resulted in a luteolin yield of 1.65 mg/g, deviating by only 0.61 % from the predicted value (relative error < 3 %), thus confirming the excellent predictive capability of the model.

### 3.5 DFT calculation

#### 3.5.1 IGMH analysis

**Fig. 6:**
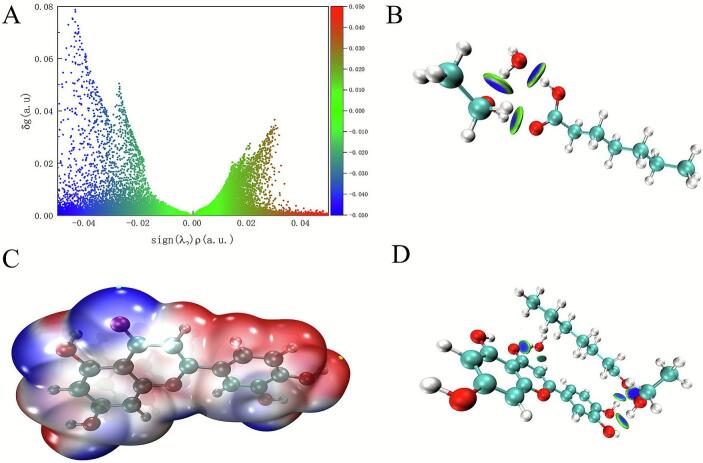
(A) The sign(λ_2_) ρ colored IGMH scatter map among the SUPRAS. (B) Isosurfaces among the SUPRAS. (C) ESP-mapped molecular VMD surface of luteolin. (D) The sign(λ_2_) ρ colored isosurfaces between luteolin and SUPRAS.

The refined IGMH was employed to elucidate the characteristics of non-covalent interactions, offering substantial improvements over the conventional IGM by incorporating real-space electron density and chemical environment corrections [41]. As shown in Fig. 6A, the three-dimensional isosurface analysis using the sign(λ_2_)ρ parameters revealed distinct regimes of interaction: blue regions (sign(λ_2_)ρ < -0.02 a.u.) correspond to strong attractive forces (e.g., hydrogen bonding), green zones (−0.02 ≤ sign(λ_2_)ρ ≤ 0.02 a.u.) indicate van der Waals interactions, and red areas (sign(λ_2_)ρ > 0.02 a.u.) represent steric repulsion [42].

As illustrated in Fig. 6B, the isosurfaces between Hea, ethanol, and water (i.e., between Hea and ethanol, Hea and water, as well as ethanol and water) are depicted in blue and green, with the peak values of δg (λ_2_) ρ (−0.04 and −0.05) being approximately 0.078. This suggests that a hydrogen-bond network is formed among heptanoic acid, ethanol, and water [43], which is consistent with the previous infrared spectroscopy results. Furthermore, to investigate the extraction mechanism of luteolin from peanut shells using supramolecular solvents, the electrostatic surface potential (ESP) distribution of the luteolin molecule was analyzed to identify the potential reaction sites (Fig. 6C). Red and blue regions represent positive and negative electrostatic potentials, respectively. The extremum points (marked in blue and yellow) on the MEP surfaces were identified as preferential sites for intermolecular binding interactions [44]. The Fig. 6D demonstrates that the RDG isosurface between luteolin and SUPRAS exhibits a similar blue-green color, further confirming the hydrogen bond-dominated interaction mode [43]. The consistent multi-scale characterization results suggest that the cooperative effect of hydrogen bonding is the primary mechanism underlying the efficient enrichment of luteolin from peanut shells using SUPRAS.

#### 3.5.2 Molecular orbital analysis

**Fig. 7:**
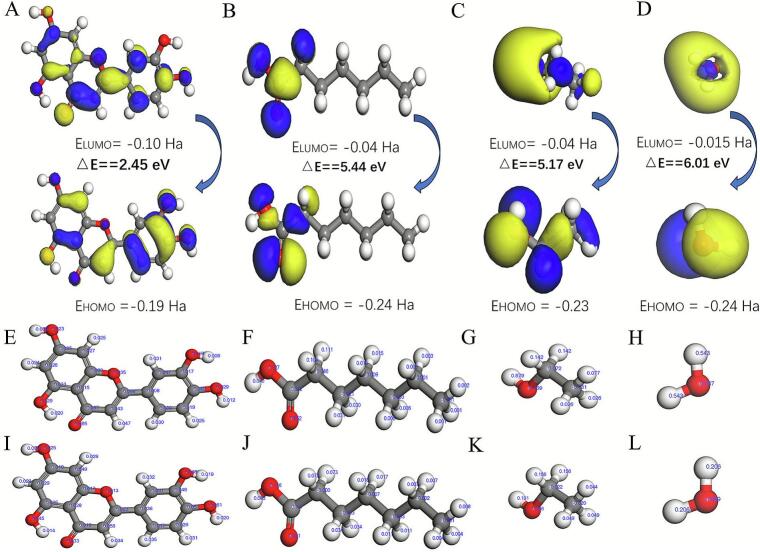
The molecular frontier orbital analysis of luteolin (A), Hea (B), ethanol (C), water (D) and distribution of Fukui Function Values for luteolin, Hea, ethanol, and water: f^+^ Distributions (E), (F), (G), (H) and f^-^ Distributions (I), (J), (K), (L).

Molecular orbital analysis (Fig. 7A-D) integrated with Fukui function quantification (Fig. 7E-L) revealed that the oxygen atom of the phenolic hydroxyl group in luteolin predominantly localizes in HOMO orbitals (f^−^=0.065), functioning as an electron donor to engage with the hydrogen atom of the hydroxyl group (O-H, f^+^=0.07) in Hea, the hydrogen atom of the hydroxyl group (–OH, f^+^=0.0879) in ethanol, and the hydrogen atom of the hydroxyl group (O-H, f^+^=0.543) in water within the SUPRAS system, thereby constructing hydrogen-bond networks [45]. Concurrently, the hydrogen atoms on the benzene ring and phenolic hydroxyl group of luteolin localize in LUMO orbitals, participating in reverse hydrogen-bond association by accepting protons from the hydroxyl groups of Hea, ethanol, and water. Energy gap analysis further demonstrated a substantial energy gap disparity between luteolin (ΔE = 2.45 eV) and the solvent components (Hea ΔE = 5.44 eV; ethanol ΔE = 5.17 eV; water ΔE = 6.01 eV), with the narrower bandgap of luteolin thermodynamically favoring the formation of stable intermolecular complexes through enhanced electronic coupling [46]. This mechanistic interpretation aligns with the hydrogen-bond-dominant interaction patterns identified via IGMH analysis.

#### 3.5.3 Analysis of intermolecular interaction energy

**Fig. 8:**
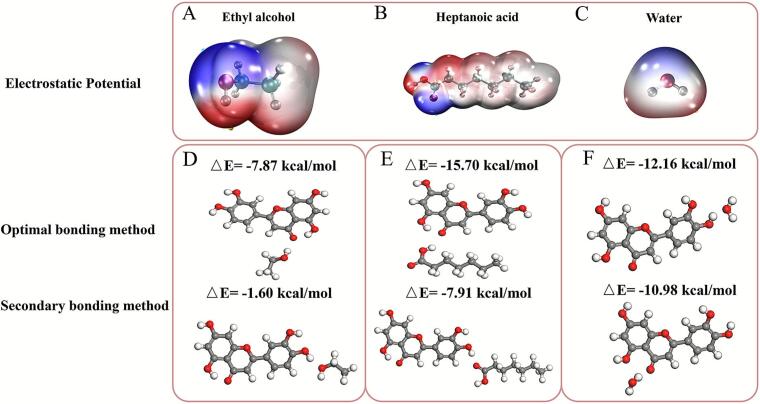
The electrostatic potential field of (A) ethanol, (B) heptanoic acid (Hea), (C) water, (D) ethanol with luteolin, (E) Hea with luteolin, (F) water with luteolin.

To further elucidate the extraction mechanism of SUPRAS, this study employed quantum chemical computational methods to characterize the interactions between luteolin and the solvent components, including water. Fig. 8A-C present the electrostatic surface potential distribution of the solvent molecules (ethyl alcohol, heptanoic acid, and water). Based on the most probable binding sites on the molecular surface, two energetically favorable binding configurations (Fig. 8D-F) were optimized through simulations [47]. The results indicate that all stable binding configurations exhibit O-H·O and O·H-O type hydrogen bond interactions, involving not only ethyl alcohol and heptanoic acid but also water. This confirms that the SUPRAS systems are formed through hydrogen bonding interactions among ethyl alcohol, heptanoic acid, and water, thus enabling efficient extraction. This conclusion is consistent with previous experimental findings and the newly supplemented mechanistic analysis of water’s role.

### 3.6 Molecular dynamics analysis

#### 3.6.1 Dynamic spatial distribution analysis

**Fig. 9:**
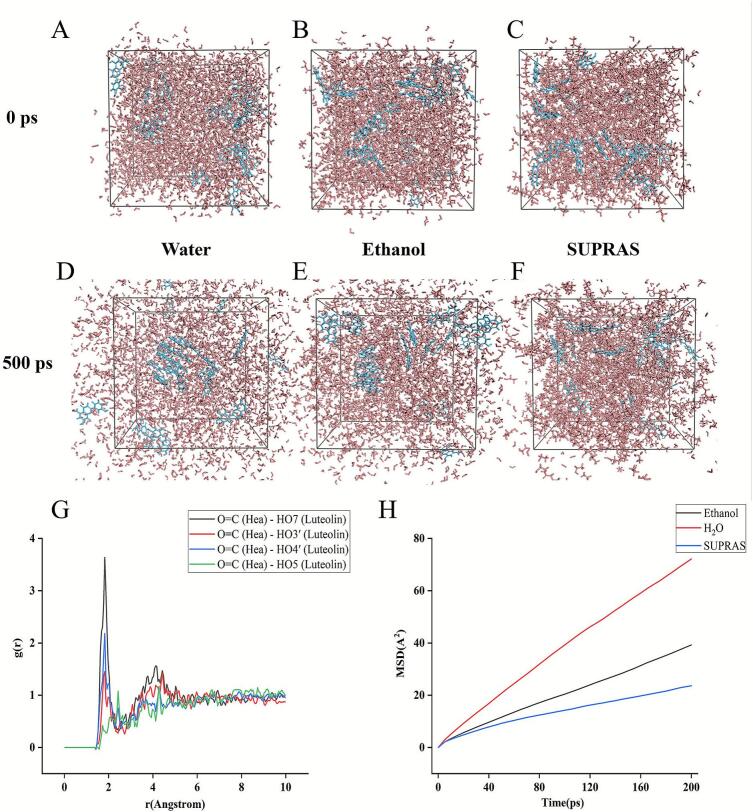
The distribution of luteolin in different solvent systems at 0 s and 100 ns was analyzed by molecular dynamics simulation (A-F). The RDF analysis of luteolin in SUPRAS solvents (G). The MSD analysis of luteolin in different solvent systems (H).

The dynamic spatial distribution characteristics of luteolin molecules in different solvent systems were systematically evaluated through molecular dynamics simulations. As depicted in Fig. 9A-F, comparative analysis of initial (0 ps) and equilibrated (500 ps) configurations across aqueous/ethanol/SUPRAS systems revealed distinct spatial heterogeneity: At the initial simulation stage (0 ps), luteolin molecules were homogeneously dispersed across all three solvent systems. Upon reaching equilibrium at 500 ps, the molecules remained homogeneously distributed in both SUPRAS and ethanol media, whereas the aqueous system displayed limited dispersion with the majority forming aggregates. This distinct morphological divergence underscores differential solute–solvent interaction patterns among the investigated systems. Given this spatial divergence, we speculate that the SUPRAS solvent system may have a larger solute–solvent contact area, thereby improving the extraction performance of the target molecule.

#### 3.6.2 RDF analysis

Radial distribution function (RDF) analysis was employed to quantitatively characterize the spatial density distribution of particles around reference molecules [48]. Characteristic peaks within the 1.5–3.5 Å range typically indicate hydrogen bonding interactions, while those in the 3.5–5.0 Å region correspond to van der Waals forces [49]. As shown in Fig. 9G, the RDF profiles between the carbonyl oxygen atoms of heptanoic acid in SUPRAS and four hydroxyl hydrogen atoms (HO (7), HO (3′), HO (4′), and HO (5)) of luteolin revealed prominent sharp peaks at 1.83 Å for the first three hydrogen atoms, with HO (5) exhibiting a distinct peak at 2.47 Å. These observations confirm the formation of strong hydrogen bonding interactions. Furthermore, discernible yet attenuated peaks within 3.5–5.0 Å confirm the synergistic involvement of van der Waals interactions. Collectively, these findings demonstrate that the SUPRAS system facilitates selective luteolin extraction through a hydrogen bond-driven mechanism with van der Waals assistance.

#### 3.6.3 MSD analysis

Mean Square Displacement (MSD) analysis quantitatively characterizes molecular diffusion by statistically evaluating temporal deviations in particle positions. The diffusion coefficient (D_i_), derived from the slope of the MSD-time curve, is defined as: $D_{i}=\frac{1}{6N}\underset{t\to \infty }{\lim}\left(d/dt\right)\Sigma_{i=1}^{N}{\left|r_{i\left(t\right)}-r_{i\left(0\right)}\right|}^{2}$[50].

As illustrated in Fig. 9H, luteolin exhibited MSD values of 72.17 Å^2^, 39.26 Å^2^, and 23.64 Å^2^ in aqueous, ethanol, and SUPRAS systems, corresponding to diffusion coefficients of 0.06 m^2^/s, 0.03 m^2^/s, and 0.01 m^2^/s, respectively. The markedly restricted molecular mobility in SUPRAS is attributed to enhanced solute–solvent interactions, which impose dynamic constraints on molecular motion[46]. This finding aligns mechanistically with prior dynamic distribution studies, collectively demonstrating that interaction intensity governs the targeted extraction efficacy of SUPRAS.

### 3.7 Comparison of traditional extraction methods

#### 3.7.1 Comparison of environment and efficiency

This study comparatively evaluated the integrated performance of ER, Water-UAE, and SUPRAS-UAE methods for luteolin extraction efficiency and environmental impact (Table 4). Experimental results demonstrate that under identical temperature conditions (70 °C), the extraction duration of SUPRAS-UAE (15 min) was merely one-eighth that of conventional ER, with significantly reduced power requirement (250 W vs 800 W). Regarding target compound recovery, SUPRAS-UAE exhibited substantial advantages, achieving a luteolin content of 1.64 mg/g − markedly higher than ER (0.95 ± 0.05 mg/g) and Water-UAE (0.61 ± 0.03 mg/g).

**Table 4:** Comparison of environmental impact and economic effect with different methods, each experiment was repeated for three times.

Parameter	ER	Water-UAE	SUPRAS-UAE
Temperature (°C)	70	70	70
Energy consumption time (min)	120	15	15
Electric power (W)	800	250	250
Yield of luteolin (mg/g)	0.95 ± 0.05	0.6 ± 0.03	1.65 ± 0.03
Electricity consumption (kW h)	1.6	0.0625	0.0625
Environmental impact (g CO2 rejected)	1280	50	50

Furthermore, based on the carbon emission factor from literature [51] (800 g CO_2_ per kWh electricity consumed), the total energy consumption of ER (0.16 kWh) resulted in 1280 g CO_2_ emissions. In contrast, SUPRAS-UAE required only 0.0625 kWh, reducing CO_2_ emissions to 50 g. These findings indicate that SUPRAS-UAE not only enhances economic viability through reduced extraction time and energy consumption but also substantially diminishes carbon footprint, validating its potential application value in green sustainable extraction technologies.

#### 3.7.2 SEM analysis

**Fig. 10:**
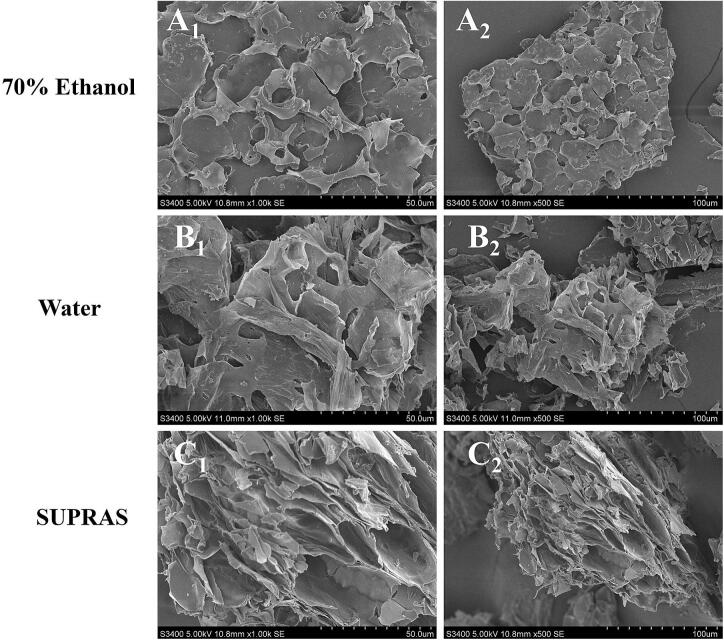
Comparison of Scanning Electron Microscopy of Luteolin Extracted by Different Methods. powder after reflux extraction with 70 % ethanol (A1: ×1000; A2: × 500;), powder after ultrasonic water treatment (B1: × 1000； B2: × 500;), and powder after ultrasonic-assisted SUPRAS extraction (C1: × 1000; C2: × 500;).

Microstructural characterization via scanning electron microscopy elucidated the mechanistic basis of extraction processes. Fig. 10A1-C2 reveals distinct morphological alterations in peanut hulls subjected to three extraction techniques: ER (70 %) induced superficial wrinkling while preserving structural integrity; Water-UAE generated porous architectures through cavitation-induced cell wall disruption; SUPRAS-UAE treatment provoked extensive tissue dissociation with aligned tubular cavities. These observations demonstrate that the SUPRAS-UAE synergy enhances cell wall permeability, facilitating compound dissolution and mass transfer kinetics. This integrated approach establishes an eco-efficient platform for targeted phytochemical recovery from botanical matrices.

### 3.8 The LC-MS analysis of peanut shell extract

**Fig. 11:**
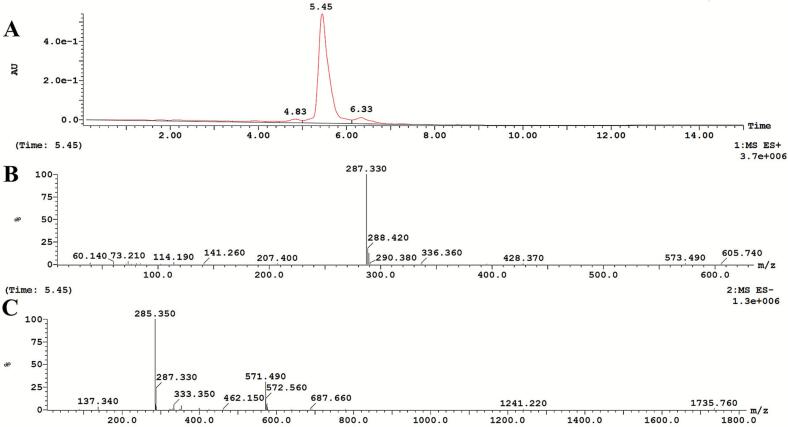
(A) Total ion chromatogram (TIC) of the peanut shell extract obtained by SUPRAS. (B) Mass spectrum (ESI^+^ mode) corresponding to the peak at 5.45 min. (C) Mass spectrum (ESI^−^ mode) corresponding to the peak at 5.45 min.

The LC-MS analysis of the peanut hull extract prepared with the supramolecular solvent revealed a predominant chromatographic peak at a retention time of 5.45 min (Fig. 11A). Mass spectrometric analysis of this major peak (Fig. 11B, C) confirmed its identification as luteolin, the target compound of this study, based on the agreement between the observed *m*/*z* of the molecular ion and the molecular weight of luteolin. It is noteworthy that only two minor chromatographic peaks with significantly smaller areas were observed on either side of the predominant luteolin peak, at retention times of 4.83 min and 6.33 min. This observation clearly indicates that the employed supramolecular solvent possesses a highly selective extraction capability for luteolin from peanut hulls, demonstrating a remarkable targeted extraction effect and enabling effective enrichment of the target product, luteolin.

## 4 Conclusion

This study presents, for the first time, a novel green extraction methodology based on a heptanoic acid–ethanol-water ternary system (SUPRAS) for the efficient extraction of luteolin from peanut shells. The proposed method is characterized by its environmentally friendly, cost-effective, and user-friendly nature. The formation mechanism of SUPRAS was characterized using Fourier-transform infrared spectroscopy (FTIR). The results elucidated a self-assembly process of reverse micelles driven by hydrogen bonding interactions among heptanoic acid molecules. Optimization of extraction conditions via Response Surface Methodology (RSM) revealed that under the following conditions: a liquid-to-solid ratio of 1:36 g/mL, a SUPRAS-to-ethanol ratio of 5:1, a temperature of 67 °C, a power of 250 W, and ultrasonic treatment for 15 min, the luteolin yield reached 1.645 mg/g. Through IGMH, molecular orbital theory calculations, interaction energy analysis, and molecular dynamics simulations, suggested that SUPRAS primarily interacts with the target compound through hydrogen bonding and other molecular interactions. In addition to the enhanced performance, the study also compares the economic feasibility and environmental impact of the SUPRAS-UAE method with traditional extraction techniques. The results demonstrate that SUPRAS-UAE not only significantly reduces extraction time and energy consumption but also improves economic viability, while substantially diminishing carbon footprint. The SUPRAS-based extraction method developed in this study offers a promising green alternative for the efficient extraction of natural products in the food, pharmaceutical, and chemical industries.

## CRediT authorship contribution statement

**Yuhan Fang:** Writing – original draft, Validation, Data curation. **Ping Zhang:** Investigation, Conceptualization. **Shuang Wang:** Software. **Lulu Li:** Visualization, Conceptualization. **Zunlai Sheng:** Writing – review & editing, Supervision.

## Declaration of competing interest

The authors declare that they have no known competing financial interests or personal relationships that could have appeared to influence the work reported in this paper.
